# Associations Between Maternal Immunisation and Reduced Rates of Preterm Birth and Stillbirth: A Population Based Retrospective Cohort Study

**DOI:** 10.3389/fimmu.2021.704254

**Published:** 2021-09-07

**Authors:** Michelle L. Giles, Mary-Ann Davey, Euan M. Wallace

**Affiliations:** The Ritchie Centre, Department of Obstetrics and Gynaecology, Monash University, Clayton, VIC, Australia

**Keywords:** immunisation, pregnancy, influenza, pertussis, preterm birth

## Abstract

Stillbirth and preterm birth (PTB) remain two of the most important, unresolved challenges in modern pregnancy care. Approximately 10% of all births are preterm with nearly one million children dying each year due to PTB. It remains the most common cause of death among children under five years of age. The numbers for stillbirth are no less shocking with 2.6 million babies stillborn each year. With minimal impact on the rate of these adverse birth outcomes over the past decade there is an urgent need to identify more effective interventions to tackle these problems. In this retrospective cohort study, we used whole-of-population data, to determine if maternal immunization during pregnancy against influenza and/or pertussis, is associated with a lower risk of PTB, delivering a small-for-gestational age (SGA) infant, developing preeclampsia or stillbirth. Women with a singleton pregnancy at 28 or more weeks’ gestation delivering in Victoria, Australia from July 2015 to December 2018 were included in the analysis. Log-binomial regression was used to measure the relationship between vaccination during pregnancy against influenza and against pertussis, with preterm birth, SGA, preeclampsia and stillbirth. Variables included in the adjusted model were maternal age, body mass index, first or subsequent birth, maternal Indigenous status, socio-economic quintile, smoking, public or private maternity care and metropolitan or rural location of the hospital. Women who received influenza vaccine were 75% less likely to have a stillbirth (aRR 025; 95% CI 0.20, 0.31), and 31% less likely to birth <37 weeks (aRR 0.69; 95% CI 0.66, 0.72). Women who received pertussis vaccine were 77% less likely to have a stillbirth (aOR 0.23; 95% CI 0.18, 0.28) and 32% less likely to birth <37 weeks gestation (aRR 0.68; 95% CI 0.66, 0.71). Vaccination also reduced the odds of small for gestational age by 13% and reduced the odds of pre-eclampsia when restricted to primiparous women. This association was seen over four different influenza seasons and independent of the time of year suggesting that any protective effect on obstetric outcomes afforded by maternal vaccination may not be due to a pathogen-specific response but rather due to pathogen-agnostic immune-modulatory effects.

## Introduction

Maternal immunisation is an established strategy to reduce the morbidity and mortality of pregnant women, and their newborn infants through transplacental transfer of pathogen specific IgG antibodies (1). In 1988, the World Health Organization (WHO) estimated that 787,000 newborns worldwide died of tetanus (2), calling for maternal and neonatal tetanus elimination (MNTE). Routine immunisation of pregnant women with tetanus toxoid containing vaccine was a key component of MNTE, together with better birth and umbilical cord care hygiene. By 2015, the WHO estimated that there had been a 96% reduction in neonatal mortality from tetanus (3). This was the first immunisation programme specific to pregnant women to be recommended globally (2). It marked the beginning of maternal immunisation being adopted as an approach to saving maternal and infant lives, particularly in low-resource settings.

Whilst in some countries, such as the United States, maternal influenza vaccination has been recommended since the 1950s, it was not until the H1N1 pandemic in 2009 that coverage rates increased (4). Reflecting that pregnant women are at higher risk of serious morbidity and mortality from influenza than the general population, in 2012 the WHO identified pregnant women as a priority population for seasonal influenza vaccination (5). Similarly, maternal vaccination with a diphtheria-tetanus-acellular pertussis vaccine (dTpa) has become standard care in many countries, including the UK, USA and Australia (6–8), reducing infant deaths from pertussis by 95% (9).

Some authors have suggested that maternal influenza vaccination may be protective against adverse pregnancy outcomes such as preterm birth (PTB), small for gestational age (SGA) and stillbirth (10–13), but others have not confirmed this finding (14, 15). There are likely several explanations for these different findings, including varying study designs and settings, different influenza seasons and vaccine matching, and variable capability in accurately measuring gestational age. However, if maternal influenza vaccination was protective against PTB and stillbirth then it would be an effective public health measure against two adverse pregnancy outcomes that have been stubbornly resistant to improvement (16, 17). Whether maternal diphtheria-tetanus-pertussis vaccination in combination with influenza vaccination has any protective effect on these pregnancy outcomes has not been reported.

Unlike previous studies we set out to determine if maternal immunisation, influenza and/or diphtheria-tetanus-pertussis, is associated with a lower risk of PTB, SGA, preeclampsia or stillbirth using whole-of-population data including with mandatory documentation of vaccination status and reliable capture of gestational age.

## Methods

We conducted a population based retrospective cohort study using data on all singleton births in Victoria at 28 or more weeks’ gestation from July 2015 to December 2018. Attending clinicians, usually midwives, provide data on maternal socio-demographic characteristics, pre-existing medical conditions, reproductive history, complications of pregnancy, procedures, details of the labour and birth, maternal morbidity, and neonatal details and morbidity on all births to the Consultative Council on Obstetric and Paediatric Mortality and Morbidity (CCOPMM). This data is captured during pregnancy and forms the Victorian Perinatal Data Collection (VPDC). Maternal vaccination against pertussis and influenza (yes/no/not known) were added to the VPDC as mandatory items for all births from 1 July 2015. In Australia the nationally funded vaccine against pertussis is the diphtheria-tetanus-pertussis vaccine and will be referred to as ‘pertussis’ vaccine for the remainder of this manuscript. Influenza vaccine is also recommended and funded by the government for all women in every pregnancy.

We excluded births before 28 weeks because, until very recently, it has been common in Victoria for women to be offered pertussis containing vaccination only from 28 weeks onwards. We also excluded multiple pregnancy because of the association between multiple pregnancy and higher rates of preterm birth and stillbirth.

The exposures of interest were vaccination against influenza, and/or pertussis during the current pregnancy. The control group chosen was women who received no vaccination at all (as the focus was on the non-specific effects of vaccination). A sensitivity analysis was performed comparing influenza vaccine compared with no influenza vaccine (regardless of pertussis status meaning women in either group may have received pertussis vaccine) and a similar analysis for pertussis vaccine *versus* no pertussis vaccine (regardless of influenza status meaning women in either group may have received an influenza vaccine). The outcomes of interest were PTB <37 weeks, <34 weeks and <32 weeks, stillbirth, SGA (defined as a birthweight <10^th^ centile for gestation and sex) and pre-eclampsia.

We included potential confounding factors for preterm birth and stillbirth in the analysis: maternal age, body mass index (BMI), parity, smoking, maternal region of birth, socioeconomic status, onset of labour, method of birth, public *versus* private admission for the birth, maternal Indigenous status and previous stillbirth.

### Analyses

We compared proportions of women with these characteristics who were vaccinated against pertussis and against influenza using the chi square test. A p-value <0.05 was considered statistically significant. We generated new variables to represent birth during the local influenza season (April to September) and non-influenza season (October to March); spontaneous onset of labour *vs* medically initiated birth (induction of labour or pre-labour caesarean section). Maternal country of birth was classified according to the Standard Australian classification of countries (SACC) 2016. For births in 2015 and 2016 the SACC 2011 was used. Maternal residential address was used to classify socio-economic status according to the Index of Relative Social Disadvantage for statistical Area 1 (a census district of approximately 400 people). This was determined by the Australian Bureau of Statistics census in 2011 for births in 2015 and 2016, and for births in 2017 and 2018 the 2016 Australian Bureau of Statistics census was used.

We used log-binomial regression to measure the relationship between vaccination during pregnancy against influenza and against pertussis, with PTB (<37 weeks, <34 weeks and <32 weeks), wfi 2SGA, preeclampsia and stillbirth. Not vaccinated with either vaccine was the reference category. Results are reported as unadjusted and adjusted Risk Ratios with their 95% confidence intervals.

Variables included in the adjusted models were those significantly associated with vaccination in the univariate analysis and known *a priori* to be associated with PTB, SGA, preeclampsia and stillbirth. They were maternal age group (in 5 year categories), maternal body mass index (BMI) category, first or subsequent birth, maternal Indigenous status, socio-economic quintile, smoking during pregnancy (yes/no/not known), public or private maternity care and metropolitan or rural location of the hospital. Analyses of stillbirth also adjusted for any prior stillbirth. Analyses of preeclampsia were restricted to primiparous women.

To interrogate the consistency of the results, sub-group analyses were planned for the most socially disadvantaged and the most advantaged quintiles of women, women who smoked in pregnancy, women born outside Australia, obese women, young women, women attending rural hospitals, those receiving private maternity care, those giving birth during influenza season and outside influenza season, and those who had a spontaneous onset of labour.

## Results

A total of 269,493 women, with a singleton pregnancy >28 weeks in Victoria gave birth between July 2015 and the December 31, 2018 and are included in our analysis. **Table 1** summarises the demographic details of the women. 138,698 (51.5%) women received influenza vaccination and 192,487 (71.4%) received pertussis vaccination during their pregnancy.

**Table 1:** Maternal characteristics (includes those with missing vaccination status; excludes <28w and multiples).

		n	%
**Vaccinated during pregnancy against influenza**			
	Yes	138698	51.5
	Not reported/inadequately described	18640	6.8
**Vaccinated during pregnancy against pertussis**			
	Yes	192487	71.4
	Not reported/inadequately described	20033	7.5
**Vaccinated during pregnancy against influenza and pertussis**			
	Yes	124140	46.10
	No/not known	145353	53.90
**First birth**			
	Yes	117395	43.6
	Not stated	3	0.0
**Maternal age group**			
	Younger than 20 years	3684	1.4
	20-24 years	24933	9.3
	25-29 years	68764	25.5
	30-34 years	102527	38.0
	35-39 years	56683	21.0
	40-44 years	11975	4.4
	45+ years	897	0.3
	Not reported/inadequately described	30	0.0
**BMI group**			
	<18.5	8106	3.0
	18.5-<25	133720	49.6
	25-<30	70807	26.3
	30-<35	31349	11.6
	35-<40	13225	4.9
	40+	8027	3.0
	Not reported/inadequately described	4259	1.6
**Maternal Indigenous status**			
	Aboriginal	3839	1.4
	Non-Aboriginal	264821	98.3
	Not reported/inadequately described	833	0.3
**Socio-economic status**			
	Most disadvantaged	53308	19.8
	2	53215	19.8
	3	53387	19.8
	4	53098	19.7
	Least disadvantaged	52900	19.6
	Not reported/inadequately described	3585	1.3
**Maternal region of birth**			
	Australia	165153	61.6
	Americas	3771	1.4
	North Africa and the Middle East	9725	3.6
	North-East Asia	14154	5.3
	North-West Europe	7625	2.8
	Oceania and Antarctica	7657	2.9
	South-East Asia	17766	6.6
	Southern and Central Asia	31392	11.7
	Southern and Eastern Europe	4976	1.9
	Sub-Saharan Africa	6021	2.2
	Not reported/inadequately described	1253	.5
**Location of hospital**			
	Metro	208403	77.3
	Rural	61090	22.7
**Smoking during pregnancy**			
	None	235343	87.3
	Smoked at all	23142	8.6
	Not reported/inadequately described	11008	4.1
**Onset of labour**			
	Spontaneous and not augmented	85668	31.8
	Induced	88435	32.8
	Spontaneous and augmented	36623	13.6
	No labour	58764	21.8
	Not reported/inadequately described	3	0.0
**Method of birth**			
	Unassisted vaginal	135155	50.2
	Assisted vaginal (forceps or vacuum)	42201	15.7
	Planned pre-labour Caesarean section	45586	16.9
	Unplanned in-labour Caesarean	31435	11.7
	Planned in-labour Caesarean section	1920	0.7
	Unplanned pre-labour Caesarean	13180	4.9
	Not reported/inadequately described	16	0.0
**Admission status**			
	Public	200636	74.5
	Private	68803	25.5
	Not reported/inadequately described	54	0.0
**Year**			
	2015	39216	14.6
	2016	77805	28.9
	2017	76669	28.5
	2018	75803	28.1
		269493	

**Table 2** summarises the proportion of women vaccinated against either influenza or pertussis according to maternal characteristics. The uptake of influenza vaccination was lower in younger women, those with a low BMI, smokers, the most disadvantaged and those who identified as Indigenous. Higher uptake of influenza and pertussis vaccination was reported in women during their first pregnancy.

**Table 2:** Proportion of women vaccinated according to maternal characteristics.

		Influenza vaccine given					Pertussis vaccine given				
		Yes		No		p-value*	Yes		No		p-value*
		n	%	n	%		n	%	n	%	
**First birth**						<0.001					<0.001
	Yes	65327	59.7	44146	40.3		89073	81.9	19712	18.1	
	No	73371	51.9	68009	48.1		103414	73.6	37160	26.4	
**Maternal age group**						<0.001					<0.001
	Younger than 20 years	1567	45.3	1892	54.7		2564	74	901	26	
	20-24 years	11290	48.5	11983	51.5		17760	75.9	5656	24.2	
	25-29 years	34608	53.8	29757	46.2		50680	78.7	13732	21.3	
	30-34 years	54736	57.4	40658	42.6		74027	78.2	20609	21.8	
	35-39 years	29897	56.9	22625	43.1		38878	75.1	12898	24.9	
	40-44 years	6135	55.7	4875	44.3		7985	73.7	2856	26.3	
	45+ years	462	56.6	354	43.4		589	73.7	210	26.3	
**BMI group**						<0.001					<0.001
	<18.5	3833	51.6	3602	48.5		5366	73	1981	27	
	18.5-<25	69870	56.2	54430	43.8		95142	77.3	27941	22.7	
	25-<30	36840	55.5	29524	44.5		51688	78.2	14427	21.8	
	30-<35	15963	54.1	13525	45.9		23085	78.3	6410	21.7	
	35-<40	6589	53.1	5830	46.9		9610	77.1	2849	22.9	
	40+	4016	53.7	3461	46.3		5694	76	1800	24	
**Maternal Indigenous status**						<0.001					<0.001
	Indigenous	1656	45.0	2025	55.0		2571	69.9	1107	30.1	
	Not Indigenous	136799	55.5	109774	44.5		189495	77.3	55585	22.7	
**Socio-economic status**						<0.001					<0.001
	Most disadvantaged	25398	51.6	23824	48.4		37019	74.9	12411	25.1	
	2	26845	53.9	22923	46.1		39005	78.4	10748	21.6	
	3	27710	55.4	22321	44.6		38849	78.1	10909	21.9	
	4	28391	57.5	20988	42.5		38308	78.2	10675	21.8	
	Least disadvantaged	28693	58.4	20456	41.6		36974	76.8	11201	23.3	
**Maternal region of birth**						<0.001					<0.001
	Australia	85903	55.2	69848	44.9		121769	78.9	32573	21.1	
	Americas	1981	56.5	1525	43.5		2652	76.6	809	23.4	
	North Africa and the Middle East	4042	45.8	4783	54.2		5569	63.2	3244	36.8	
	North-East Asia	6978	54.4	5845	45.6		9310	72.7	3504	27.4	
	North-West Europe	4268	60.0	2892	40.4		5688	80.4	1385	19.6	
	Oceania and Antarctica	3501	49.8	3527	50.2		5008	71.3	2013	28.7	
	South-East Asia	9391	58.8	6580	41.2		12103	75.9	3842	24.1	
	Southern and Central Asia	17405	60.5	11346	39.5		22858	79.1	6025	20.9	
	Southern and Eastern Europe	2120	46.8	2411	53.2		3282	72.6	1240	27.4	
	Sub-Saharan Africa	2625	48.2	2827	51.9		3570	65.8	1860	34.3	
**Smoking during pregnancy**						<0.001					<0.001
	None	123418	56.4	95252	43.6		169219	77.9	47977	22.1	
	Smoked at all	9497	43.6	12276	56.4		15718	72.2	6049	27.8	
**Admission status**						<0.001					<0.001
	Public	102875	54.5	86037	45.5		151963	80.0	38121	20.1	
	Private	35808	57.9	26094	42.2		40508	68.4	18729	31.6	
**Location of hospital**											<0.001
	Metropolitan area	107277	56.2	83570	43.8	<0.001	142457	75.2	46947	24.8	
	Rural area	31421	52.4	28585	47.6		50030	83.4	9926	16.6	

*Chi square for vaccinated vs not vaccinated.

After adjusting for possible confounding factors including parity, maternal age, BMI, socioeconomic status, smoking, private/public admission for birth, maternal Indigenous status, maternal region of birth, metropolitan/rural location of hospital and any prior stillbirth (for stillbirth analysis only) receipt of either influenza or pertussis vaccination was associated with a significantly lower rate of PTB, FGR, stillbirth and preeclampsia for primiparous women (**Table 3**). There was little difference in the effect when the analysis was restricted to spontaneous PTB suggesting the main effect of vaccination on PTB appears to be on spontaneous PTB rate (**Table 3**). Overall, compared to women who received no vaccine, women who received both influenza and pertussis vaccine had 79% lower odds of stillbirth and 61% lower odds of PTB <32 weeks (**Table 3**).

**Table 3:** Singleton births at >=28 weeks July 2015-December 2018 (those with missing vaccination status excluded).

	Influenza vaccination *vs* neither							Pertussis vaccination *vs* neither							Both vaccines *vs* neither						
	n (%) in vaccinated group	n (%) in not vaccinated group	p-value	RR	95%CI	aRR*	95%CI	n (%) in vaccinated group	n (%) in not vaccinated group	p-value	RR	95%CI	aRR*	95%CI	n (%) in vaccinated group	n (%) in not vaccinated group	p-value	RR	95%CI	aRR*	95%CI
Stillbirth**	155 (0.11)	202 (0.46)	<0.001	0.24	(0.20, 0.30)	0.25	(0.20, 0.31)	204 (0.11)	202 (0.46)	<0.001	0.23	(0.19, 0.28)	0.23	(0.18, 0.28)	123 (0.10)	202 (0.46)	<0.001	0.22	(0.17, 0.27)	0.21	(0.16, 0.26)
Birth before 37 weeks	7679 (5.54)	3636 (8.23)	<0.001	0.67	(0.65, 0.70)	0.69	(0.66, 0.72)	10495 (5.45)	3636 (8.23)	<0.001	0.66	(0.64, 0.69)	0.68	(0.66, 0.71)	6616 (5.33)	3636 (8.23)	<0.001	0.65	(0.62, 0.67)	0.66	(0.64, 0.69)
Spontaneous (only) birth before 37 weeks	3184 (5.26)	1635 (7.67)	<0.001	0.69	(0.65, 0.73)	0.70	(0.66, 0.74)	4412 (5.11)	1635 (7.67)	<0.001	0.67	(0.63, 0.70)	0.69	(0.65, 0.73)	2728 (5.01)	1635 (7.67)	<0.001	0.65	(0.62, 0.69)	0.67	(0.63, 0.71)
Birth before 34 weeks	1290 (0.93)	953 (2.16)	<0.001	0.43	(0.40, 0.47)	0.44	(0.41, 0.48)	1747 (0.91)	953 (2.16)	<0.001	0.42	(0.39, 0.46)	0.44	(0.41, 0.48)	1037 (0.84)	953 (2.16)	<0.001	0.39	(0.35, 0.42)	0.39	(0.36, 0.43)
Birth before 32 weeks	538 (0.39)	419 (0.95)	<0.001	0.41	(0.36, 0.46)	0.43	(0.37, 0.49)	727 (0.38)	419 (0.95)	<0.001	0.40	(0.35, 0.45)	0.43	(0.38, 0.49)	424 (0.34)	419 (0.95)	<0.001	0.36	(0.31, 0.41)	0.39	(0.36, 0.43)
Pre-eclampsia	3695 (2.66)	1111 (2.51	0.087	1.06	(0.99, 1.13)	0.94	(0.87, 1.00)	5146 (2.67)	1111 (2.51)	0.06	1.06	(1.0, 1.13)	0.93	(0.87, 0.99)	3400 (2.74)	1111 (2.51)	0.012	1.09	(1.02, 1.16)	0.94	(0.87, 1.00)
Pre-eclampsia (First births only)	589 (4.07)	2448 (3.75)	0.066	0.92	(0.84, 1.01)	0.89	(0.82, 0.98)	3396 (3.81)	589 (4.07)	0.136	0.94	(0.86, 1.02)	0.89	(0.82, 0.97)	2279 (3.85)	589 (4.07)	0.227	0.95	(0.87, 1.03)	0.9	(0.82, 0.98)
Birthweight <10th centile	11399 (8.22)	4024 (9.11)	<0.001	0.90	(0.87, 0.93)	0.88	(0.85, 0.91)	15728 (8.17)	4024 (9.11)	<0.001	0.90	(0.87, 0.93)	0.87	(0.84, 0.90)	10212 (8.23)	4024 (9.11)	<0.001	0.9	(0.87, 0.93)	0.87	(0.84, 0.90)

*Adjusted for parity, maternal age, BMI group, SES, smoking during pregnancy, public/private admission for the birth, maternal Aboriginal status, Maternal region of birth, metropolitan/rural location of hospital. **also adjusted for any prior stillbirth.

To explore possible pathways to reduced PTB and stillbirth, we examined whether maternal vaccination was associated with impaired fetal growth and preeclampsia. Maternal vaccination was associated with modest reductions in the rates of both SGA and preeclampsia (**Table 3**). Women who received influenza vaccine were 13% less likely to have a baby <10^th^ centile for birthweight and 11% less likely to have preeclampsia. The reduction in preeclampsia was only significant when the analysis was restricted to primiparous women (**Table 3**).

The associations between reduced stillbirth and PTB, and maternal influenza vaccination were just as strong when analysed according to birth during or outside of influenza season (**Table 4**) and across different years (**Table 5**), irrespective of the severity of the influenza season.

**Table 4:** Effect of influenza vaccination on obstetric outcomes by birth during *versus* outside of influenza season.

Flu season (April to September)		n(%) in those vaccinated against influenza	n(%) in those not vaccinated against influenza or pertussis	p-value	aRR	95% CI
Stillbirth		78 (0.11)	100 (0.46)	<0.001	0.23	(0.17, 0.32)
Birth before 37 weeks		4231 (5.86)	1745 (8.05)	<0.001	0.75	(0.71, 0.80)
Birth before 34 weeks		727 (1.01)	445 (2.05)	<0.001	0.51	(0.45, 0.57)
Birth before 32 weeks		293 (0.41)	191 (0.88)	<0.001	0.50	(0.41, 0.60)
Pre-eclampsia		1964 (2.72)	579 (2.67)	0.693	0.89	(0.81, 0.98)
Birthweight <10th centile		5941 (8.23)	2009 (9.28)	<0.001	0.88	(0.84, 0.92)
**Not Flu season (October to March)**						
Stillbirth		77 (0.12)	102 (0.45)	<0.001	0.26	(0.17, 0.36)
Birth before 37 weeks		3448 (5.18)	1891 (8.40)	<0.001	0.63	(0.59, 0.66)
Birth before 34 weeks		563 (0.85)	508 (2.26)	<0.001	0.38	(0.34, 0.43)
Birth before 32 weeks		245 (0.37)	228 (1.01)	<0.001	0.37	(0.30, 0.45)
Pre-eclampsia		1731 (2.60)	532 (2.36)	0.049	0.98	(0.89, 1.08)
Birthweight <10th centile		5458 (8.20)	2015 (8.95)	<0.001	0.88	(0.84, 0.93)

**Table 5:** Outcome according to year.

	2015		2016		2017		2018	
	aRR	95%CI	aRR	95%CI	aRR	95%CI	aRR	95%CI
Stillbirth	0.38	(0.21, 0.70)	0.16	(0.10, 0.27)	0.27	(0.18, 0.41)	0.18	(0.12, 0.29)
Birth<37 weeks	0.71	(0.65, 0.79)	0.69	(0.64, 0.75)	0.62	(0.57, 0.67)	0.64	(0.59, 0.70)
Birth <34 weeks	0.54	(0.43, 0.69)	0.43	(0.36, 0.51)	0.35	(0.30, 0.42)	0.40	(0.33, 0.48)
Birth <32 weeks	0.55	(0.38, 0.81)	0.42	(0.32, 0.54)	0.29	(0.22, 0.37)	0.41	(0.30, 0.55)

Sub-group analyses were largely consistent with the overall results though some of the relationships were stronger including in younger women, women who smoked in pregnancy and those attending rural hospitals, and weaker in women receiving private maternity care. Similarly, sensitivity analyses comparing receipt of influenza vaccine *versus* no vaccine (regardless of pertussis status) and receipt of pertussis vaccine *versus* no pertussis vaccine (regardless of influenza status) were largely consistent, although the magnitude of protection was slightly greater for pertussis vaccine (**Supplementary Table**).

## Discussion

This is the largest study published to date exploring the association between maternal immunisation with influenza and pertussis vaccines and the pregnancy outcomes of PTB, preeclampsia, giving birth to a SGA infant and stillbirth. We found that influenza vaccination and pertussis vaccination during pregnancy were both associated with lower rates of PTB, including very PTB (<32 weeks gestation) and stillbirth. Given the persistently high rates of PTB and stillbirth globally, the potential implications for low, middle- and high-income settings are significant.

We wished to explore possible effects of vaccination on preeclampsia and impaired fetal growth because they are both on the causal pathways to preterm birth and stillbirth (18, 19). While vaccination was associated with reductions in both preeclampsia and SGA, the aRRs for each were more modest than those for PTB and stillbirth. Together with the late pregnancy timing of vaccination, this suggests to us that any mechanisms of action may not be *via* improved placentation. Closer examination of classifications of stillbirth and/or timing of vaccination in pregnancy may provide further clues to possible mechanisms although this data was not available at the time of our study.

The association between maternal vaccination and improved obstetric outcomes was afforded by either vaccination, was relatively consistent across years irrespective of differences in influenza activity, and was present outside of influenza season. This suggests that any underlying mechanism(s) of protection may not be pathogen-specific.

### Interpretation

We suggest that maternal vaccination may be associated with improved pregnancy outcomes *via* pathogen-agnostic immune-modulatory effects. Pregnancy itself is a state of heightened inflammation (20) but PTB, particularly spontaneous PTB, and preeclampsia are conditions characterized by excessive and progressive systemic maternal inflammation (18–21). We suggest that maternal vaccination may modify the maternal immune trajectory, reducing harmful systemic inflammation such that pregnancy outcomes are improved. The suggestion that vaccines impact the host beyond the pathogen-specific immune response is not new (22). For example, neonatal BCG induces a rapid onset granulopoiesis that protects newborns from non-TB sepsis (23). It is this observation that prompted researchers to consider BCG vaccine to protect against and/or modify the clinical response to SARS-CoV-2 infection. Indeed, vaccination-induced pathogen-agnostic collateral immune effects are just beginning to be recognized more widely as promising approaches to improve neonatal health (24).

### Strengths

Strengths of our findings are that our data are from a whole population over multiple years, and that the accuracy of the data has been previously confirmed (25). Further, while most previously published data on obstetric outcomes is heavily weighted to the H1N1 pandemic, when a monovalent vaccine was used, our study covers a period during which trivalent and quadrivalent vaccines were in use. In comparison, the meta-analysis by Jeong and colleagues (14), which did not report a significant association, included several observational studies, the largest one included 130,996 vaccinated women. Whilst acknowledging the discordance in results to a recently published pooled analysis of three randomized controlled trials (10,002 women) our study included data on a much larger population dataset (269,493 women) in a resource rich setting with accurate determination of gestational age at delivery.

### Limitations

Given the observational nature of our study, we cannot exclude confounding although an attempt to control for this was made by including many potential confounding factors from the perinatal data system. In addition, we were only able to control for socioeconomic status by an area-level summary statistic for a woman’s census district, rather than individual-level data on family income. A further limitation of our data is the lack of information on the timing of vaccination during pregnancy. The VPDC does not collect date of vaccination therefore analysis according to timing of exposure (vaccination) could not be done. Immortal time bias refers to a period of follow-up during which, by study design the outcome cannot occur. In pregnancy studies, this means that the protective effect of the intervention may be overestimated as the shortened pregnancy duration association with adverse fetal outcomes limits the opportunity to be exposed. When considering studies exploring the risk or benefit of vaccination, it necessarily biases the results in favour of the treatment by conferring a spurious advantage to the treatment group. In previously published studies however, even when accounting for this, some associations remain (26).

### Implications

Irrespective of mechanisms, every year 2.6 million babies are stillborn around the world (16). Referred to as the ‘silent epidemic’, there has been a worldwide call to action to address this. In 2014, the *Every Newborn Action Plan* proposed a global target of reducing the stillbirth rate to ten per 1000 births or less in every country by the year 2035 (27). Similarly, an estimated 15 million babies are born preterm annually around the world, with 80% of these being born in sub-Saharan Africa and Asia (28). Prematurity is the world’s single biggest cause of newborn death, and the second leading cause of all child deaths, after pneumonia. In many countries the rate of PTB is actually increasing (28), highlighting the urgent need to find new strategies that address both of these global health issues. If our findings represent a causal relationship, then there may be opportunities to finally make a significant impact in reducing these adverse obstetric outcomes that have been stubbornly resistant to interventions (29).

Critically, many of the countries with the highest rates of stillbirth and PTB are yet to add influenza or pertussis vaccine to their maternal immunisation programme. In 2014, only 115 of 194 countries (59%) had a national influenza policy and, of these, fewer than half included pregnant women (30). The addition of maternal vaccines beyond tetanus toxoid containing vaccine in low- and middle-income countries has been slow (31). Likely reasons for this include the cost and/or challenges with prioritization over other healthcare expenses. Further research is required across all income settings, to better understand the immune trajectory of pregnancy and how maternal immunisation may impact this immune trajectory and other health outcomes. Any non-specific vaccine effects may ultimately provide further health economic justification for considering the addition of maternal vaccines in national immunisation programmes.

## Conclusion

While the initial causes of PTB and stillbirth are clearly complex and diverse, many are a spectrum of one underlying immune mediated problem. Our observation that maternal immunisation may be associated with reduction in the incidence of stillbirth, PTB and SGA offers hope that we can purposefully harness and manipulate maternal immunology to improve pregnancy outcomes. Our results have also identified important areas for future research, such as the need to understand the immunological effects of vaccination during pregnancy, by type of vaccination, number and timing of vaccination, and to explore this in relation to the pathophysiology of PTB and stillbirth. Such insights may shed light on new opportunities to prevent PTB and stillbirth.

## Data Availability Statement

The datasets presented in this article are not readily available because the release of potentially identifiable information to any persons not listed in s.41 of the Public Health and Wellbeing Act is only permitted for the purpose of research. Requests to access the datasets should be directed to Consultative Council on Obstetric and Paediatric Mortality and Morbidity, Victorian Perinatal Data Collection.

## Ethics Statement

This study was approved by the Monash University Human Research and Ethics Committee. Written informed consent for participation was not required for this study in accordance with national legislation and the institutional requirements.

## Author Contributions 

MG and EW developed the concept, contributed to interpretation of the data and writing of the manuscript. M-AD performed the statistical analysis, contributed to interpretation of the data and writing of the manuscript. All authors contributed to the article and approved the submitted version.

## Author Disclaimer

The conclusions, findings, opinions and views or recommendations expressed in this paper are strictly those of the author(s). They do not necessarily reflect those of CCOPMM.

## Conflict of Interest

EW is a recipient of a National Health and Medical Research Council (NHMRC) Program Grant (APP1113902) and is a chief investigator on the NHMRC Stillbirth Centre for Research Excellence (APP1116640).

The remaining authors declare that the research was conducted in the absence of any commercial or financial relationships that could be construed as a potential conflict of interest.

## Publisher’s Note

All claims expressed in this article are solely those of the authors and do not necessarily represent those of their affiliated organizations, or those of the publisher, the editors and the reviewers. Any product that may be evaluated in this article, or claim that may be made by its manufacturer, is not guaranteed or endorsed by the publisher.
